# Effect of Sodium Chloride, Sodium Nitrite and Sodium Nitrate on the Infectivity of Hepatitis E Virus

**DOI:** 10.1007/s12560-020-09440-2

**Published:** 2020-08-27

**Authors:** Alexander Wolff, Taras Günther, Thiemo Albert, Reimar Johne

**Affiliations:** 1 German Federal Institute for Risk Assessment, Max-Dohrn-Straße 8-10, 10589 Berlin, Germany; 2 Institute of Food Hygiene, University of Leipzig, An den Tierkliniken 1, 04103 Leipzig, Germany

**Keywords:** Hepatitis E virus, Inactivation, Sodium chloride, Sodium nitrite, Sodium nitrate

## Abstract

Hepatitis E virus (HEV) infection can cause acute and chronic hepatitis in humans. The zoonotic HEV genotype 3, which is highly prevalent in Europe, is mainly transmitted by consumption of raw meat and raw meat products produced from infected pigs or wild boars. High salt concentrations represent an important measure to preserve meat products and to inactivate foodborne pathogens. Here, an HEV preparation in phosphate-buffered saline (PBS) was subjected to different salt concentrations and the remaining infectivity was measured in a cell culture assay. Treatments with up to 20% sodium chloride for 24 h at 23 °C, with and without addition of 0.015% sodium nitrite or 0.03% sodium nitrate, did not lead to virus inactivation as compared to PBS only. Conditions usually applied for short-term and long-term fermented raw sausages were simulated by incubation at 22 °C for up to 6 days and at 16 °C for up to 8 weeks, respectively. Only 2% sodium chloride with 0.015% sodium nitrite showed a weak (< 1 log_10_), but significant, infectivity reduction after 2 and 4 days as compared to PBS only. Addition of 2% sodium chloride and 0.03% sodium nitrate showed a slight, but not significant, decrease in infectivity after 2 and 8 weeks as compared to PBS only. In conclusion, HEV is highly stable at high salt concentrations and at salt conditions usually applied to preserve raw meat products.

Infection with the hepatitis E virus (HEV) can cause acute or chronic hepatitis in humans. Especially, pregnant women or patients with underlying liver diseases are at risk of severe courses of acute hepatitis. In addition, chronic infections with liver cirrhosis and extrahepatic manifestations such as neurologic disorders have been described in immunosuppressed patients (Narayanan et al. 2019). Steeply increasing numbers of notified hepatitis E cases have been recently reported in several European countries (Aspinall et al. 2017). HEV is a single-stranded RNA virus, which can form quasi-enveloped and non-enveloped particles. Both particle types are infectious in cell culture (Yin et al. 2016). Most human-pathogenic HEV strains can be classified into the genotypes 1 to 4 (Johne et al. 2014). Among those, genotypes 3 and 4 are zoonotic and prevalent in reservoir animals, such as wild boars and pigs (Pavio et al. 2017). The most important transmission route of these zoonotic genotypes is considered to be via consumption of undercooked meat or raw meat products from infected animals, which is supported by several case reports (Colson et al. 2010; Masuda et al. 2005; Matsuda et al. 2003). However, the distinct risk of infection by specific meat products is unknown so far. One reason for this is that the stability of HEV under different conditions used for food production and preservation is not fully understood. Salting of meat to reduce the water activity value is an important measure for preserving food, among other microbial hurdles (Leistner 2000). For salting of meat, nitrite or nitrate curing salt is commonly used to influence the color and the taste as well as to enhance the shelf life and safety of meat products. Nitrite exhibits an antimicrobial activity by inhibiting enzymes or disrupting electron transports in several microbes (Wirth 1980; Mueller-Herbst et al. 2014).

The aim of this study was to assess the stability of HEV against different salt concentrations at conditions usually applied during meat preservation. Due to the lack of reliable methods for measurement of HEV infectivity directly in meat products (Cook et al. 2017), the stability of HEV against different salts was analyzed here using pure virus suspensions. The experiments were performed at different temperatures and incubation times, simulating the conditions during short-term and long-term fermentation of raw sausages.

For stability experiments and residual infectivity titrations, an established cell culture system using the human HEV genotype 3c strain 47832c was used as described (Wolff et al. 2020). Briefly, a virus stock suspension was prepared by collecting the supernatant of persistently HEV-infected A549 cells after three freeze/thaw cycles, which results in a mixture of quasi-enveloped and non-enveloped virus particles (Wolff et al. 2020). These particles were then pelleted by ultracentrifugation and the pellet was resuspended in phosphate-buffered saline (PBS, PAN-Biotech GmbH, Germany). The resulting virus stock suspension had a concentration of 2.9 × 10^4^ focus-forming units (ffu)/ml of infectious HEV particles. Salt stock solutions with varying concentrations of sodium chloride, sodium nitrite and sodium nitrate (Merck, Germany) were prepared in PBS. For the stability experiments, aliquots of 500 µl virus stock suspension were mixed with the salt stock solutions resulting in the desired salt concentrations at final volumes of up to 5 ml. All concentrations were calculated in percent by mass [m/m]. The mixtures were incubated at the indicated temperatures and time intervals. In order to exclude pH effects on HEV infectivity during the salt experiments, control salt mixtures without virus were measured with a micro pH electrode at days 0 and 7 as described (Wolff et al. 2020). At the desired time-points, each virus sample was diluted with PBS to a final volume of 20 ml to stop the incubation. Thereafter, the samples were ultrafiltrated using Vivaspin 20 ultrafiltration tubes (50 kDa MWCO, PES membrane, Sartorius, Germany) to a final volume of 500 µl and stored at 4 °C until infectivity titration on the same day, which was performed exactly as described (Johne et al. 2016). Briefly, tenfold dilutions of the samples were used for infection of A549/D3 cells in a 96-well plate format. After 2 weeks, infected cells were stained, using an HEV capsid protein-specific rabbit hyperimmune serum followed by a FITC-conjugated antirabbit IgG antibody, and visualized using an inverse fluorescence microscope. The number of fluorescence foci was counted manually and infectivity values were calculated in ffu/ml. Each experimental condition was analyzed in 2 independent biological replications with 4 technical replications each. The infectivity values were log_10_-transformed and thereafter statistically analyzed. Statistical tests as for normal distribution (Shapiro–Wilk test and q–q plots), general difference between all samples (Kruskal–Wallis test) and specific differences between individual samples (pairwise Wilcoxon test for unpaired samples) were performed in R 3.5 (R Core Team 2019). In all statistical tests the significance level was set to *α* = 5%.

In the first experiment, HEV was subjected to extreme salt concentrations for 24 h at 23 °C (Fig. 1). In detail, the HEV stock suspension was treated with sodium chloride at concentrations of 2%, 10% or 20%. As a control reaction without adding of salt, only the HEV stock suspension (HEV in PBS containing 0.8% sodium chloride) was used. Some of the samples were treated additionally with 0.015% sodium nitrite or 0.03% sodium nitrate. After the incubation time, the arithmetic mean of all incubated samples was 0.6 log_10_ ffu/ml lower than the arithmetic mean of the non-incubated sample. However, no significant differences were observed between all incubated samples irrespectively of their salt concentrations. The results of the experiment indicate that HEV exhibits a very high resistance to various salt concentrations and conditions. Even at a concentration of 20% sodium chloride, which is much higher than those commonly used in food, and concentrations of sodium nitrite and sodium nitrate representing the upper limits allowed due to European legislation on food additives (Regulation (EC) No 1333/2008), the virus turned out to be stable during the short time incubation analyzed here.

**Fig. 1:**
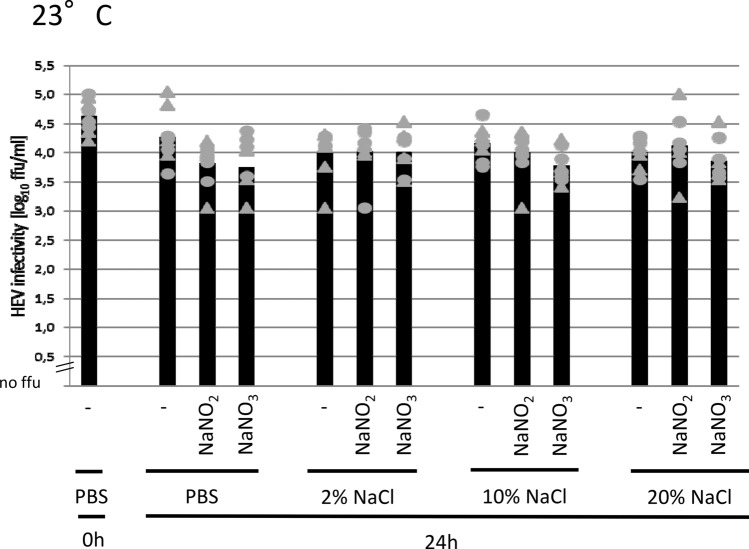
Infectivity of HEV after treatment with high salt concentrations. The samples were incubated with the indicated salt concentrations in phosphate-buffered saline (PBS) for 24 h at 23 °C. The infectious virus titers were titrated on A549/D3 cell cultures. The infectivity values, generated from two independent treatments (first treatment gray circles and second treatment gray triangles) with 4 replications each, and the arithmetic means (black columns) are indicated in log_10_ focus-forming units/ml. No significant differences were detected between all samples after 24 h. NaCl: sodium chloride at the indicated concentrations; NaNO_2_: sodium nitrite (0.015%); NaNO_3_: sodium nitrate (0.03%)

In the second experiment, HEV was subjected to salt conditions which normally occur during short-term fermentation of raw sausages (Fig. 2a). Incubation was done for up to 6 days at 22 °C which is typical for production of this sausage type (Keim and Franke 2007). In detail, the HEV stock suspension was treated with 2% sodium chloride or 2% sodium chloride with 0.015% sodium nitrite and compared to the HEV stock suspension in PBS only. The mean infectivity decreased during six days of incubation by 1.6 log_10_ ffu/ml as compared to the initial value, with a rapid loss in the first two days followed by a plateau phase. Overall, only minor differences between all conditions could be observed. Only the condition with sodium chloride and sodium nitrite at days 2 and 4 showed significantly lower mean titers as compared to the others, although the observed differences were low (< 1 log_10_ ffu/ml). In detail, significant differences were found for 2% sodium chloride with 0.015% sodium nitrite vs. PBS only for after 2 (*p* = 0.037) and 4 (*p* = 0.028) days of incubation, and for 2% sodium chloride with 0.015% sodium nitrite vs. 2% sodium chloride for day 2 (*p* = 0.028). In the pH control samples, a decrease of pH values (from a maximum of pH 7.5 to a minimum of pH 6.4) due to an increase of sodium chloride was found, whereas the effect of sodium nitrite and sodium nitrate in the mixtures was negligible. After 7 days of storage at 22 °C, all pH values decreased slightly, with an arithmetic mean pH decrease of 0.03. It has been shown recently, that only pH values < 5 had significant effects on HEV infectivity after storage for 7 days at room temperature (Wolff et al. 2020); therefore, the pH effect can be considered minimal here. In summary, the experiment showed that salt conditions commonly occurring during short-term fermentation of raw sausages have no or only very weak effects on HEV inactivation.

**Fig. 2:**
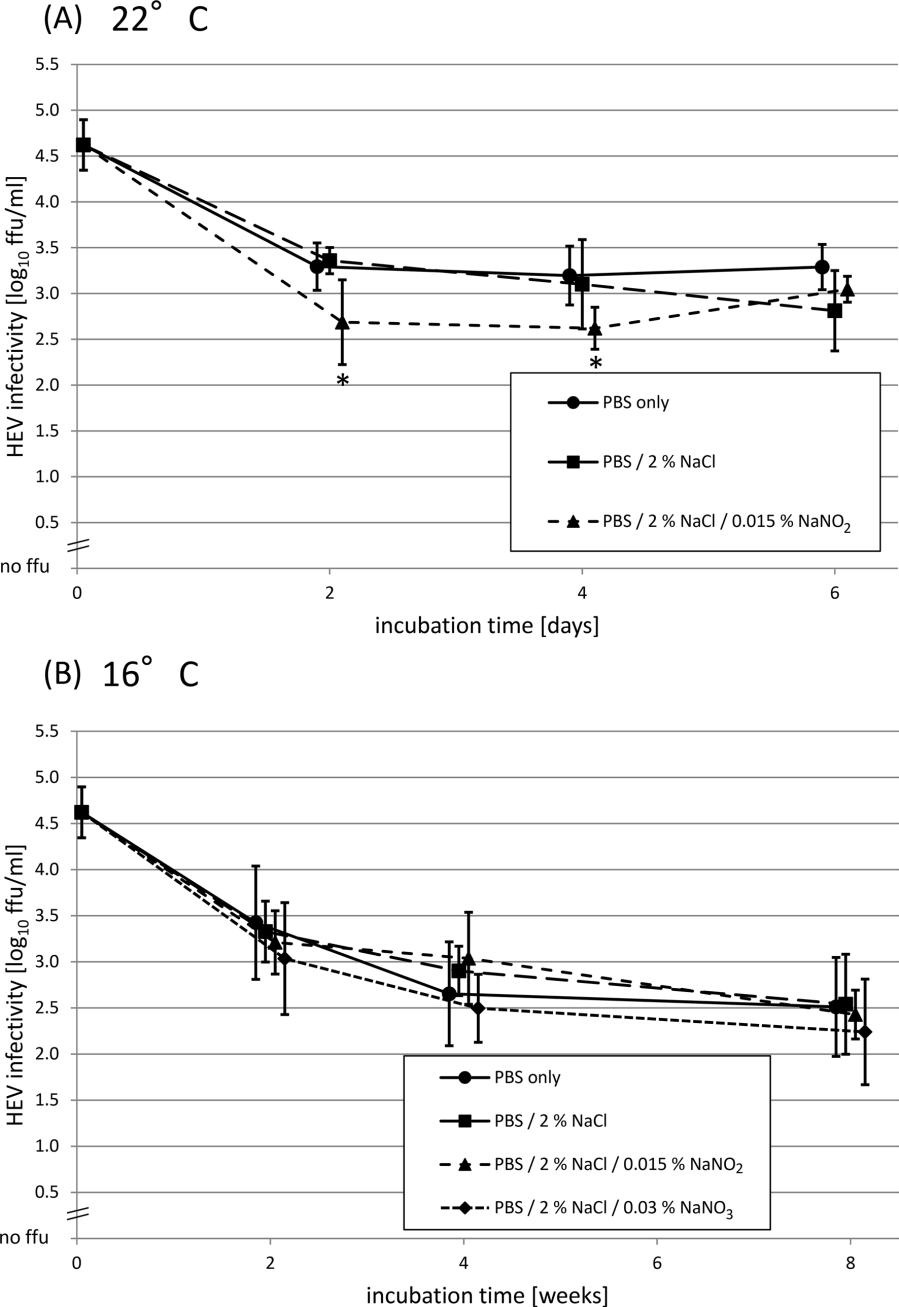
Time-courses of HEV infectivity after incubation at different salt conditions. **a** Treatment at 22 °C for up to 6 days; **b** treatment at 16 °C for up to 8 weeks. The samples were incubated with phosphate-buffered saline (PBS) only (black circles), PBS containing 2% sodium chloride (NaCl) (black squares), PBS containing 2% sodium chloride and 0.015% sodium nitrite (NaNO_2_) (black triangles) or PBS containing 2% sodium chloride and 0.03% sodium nitrate (NaNO_3_) (black diamonds). The infectious virus titers were titrated on A549/D3 cell cultures. The infectivity values shown as arithmetic means from two independent treatments with 4 replications each are indicated in log_10_ focus-forming units/ml, as well as their standard deviations. *Significant differences (*p* < 0.05)

In the third experiment, HEV was subjected to salt conditions which normally occur during long-term fermentation of raw sausages (Fig. 2b). Incubation was done for up to 8 weeks at 16 °C which is typical for production of this sausage type (Keim and Franke 2007). In detail, the HEV stock suspension was treated with 2% sodium chloride, 2% sodium chloride with 0.015% sodium nitrite or 2% sodium chloride with 0.03% sodium nitrate in comparison to the HEV stock suspension in PBS only. The mean infectivity decreased about 1.8 log_10_ ffu/ml during the whole incubation time period of 8 weeks compared to the initial value, with a rapid decrease until week 2, followed by a weaker decrease. No obvious differences between all conditions could be observed. However, a general, but not significant, trend to lower mean values of infectivity for the treatment with sodium chloride and sodium nitrate was found. Generally, it has to be concluded from this experiment that even with longer incubation times, the salt conditions have no obvious effect on HEV inactivation.

The stability of other viruses against salt conditions usually occurring during food preservation has been only scarcely analyzed so far. Enteric cytopathic human orphan (ECHO) virus, a surrogate for human enteroviruses, also shows a very high resistance against extreme concentrations of sodium chloride. After 7 days of exposure to a 20% sodium chloride solution at 4 °C or 20 °C, no inactivating effect was found (Straube et al. 2011). In contrast, the infectivity reduction of feline calicivirus (FCV), a surrogate for human noroviruses, correlated with higher sodium chloride concentrations, longer incubation times and higher temperatures. After 3 h of incubation with sodium chloride concentrations up to 20% at 4 °C or 20 °C, no significant loss of virus infectivity could be detected compared to the PBS control. However, virus titers decreased significantly after 7 days of incubation at higher temperature (20 °C), with stronger reduction by 6%, 12% and 20% sodium chloride as compared to 2% and the PBS control (Straube et al. 2011). Adding of 0.01%, 0.015% or 0.02% sodium nitrite to a 2% sodium chloride solution showed no effect on infectivity reduction of FCV or ECHO virus (Straube et al. 2011). It can be concluded from this comparison, that HEV behaves similar to ECHO virus with regard to salt stability, but FCV should therefore not be considered as surrogate for HEV in stability experiments involving salts.

In conclusion, our study showed that HEV is highly stable at different salt concentrations. The results indicate that HEV will not be efficiently inactivated at salt conditions occurring during short-term or long-term fermentation of raw sausages. Another parameter commonly used for preservation of raw sausages is lowering of the pH value. However, it has been shown recently that HEV is also highly stable against a large range of different pH values, including those usually occurring during fermentation processes (Wolff et al. 2020). Taken together, it has to be considered that residual infectious virus will still be present in fermented meat products, if sufficiently high HEV-contaminated meat was used as starting material. One limitation of the study is that the analysis of the HEV salt stability was done in a liquid solution, which may not completely reflect the situation in meat products. Future investigations should therefore focus on direct measurement of HEV infectivity in the meat matrix, in order to validate the findings of this study.
